# Knowledge Regarding Basic Facts of Stroke Among Final Year MBBS Students and House Officers: A Cross-Sectional Survey of 708 Respondents from Pakistan

**DOI:** 10.7759/cureus.539

**Published:** 2016-03-23

**Authors:** Mohammad U Khubaib, Farooq A Rathore, Ahmed Waqas, Mohsin M Jan, Sana Sohail

**Affiliations:** 1 Department of Rehabilitation Medicine, CMH Lahore Medical College and Institute of Dentistry; 2 Final Year MBBS Student, CMH Lahore Medical College and Institute of Dentistry; 3 House Job, CMH Lahore Medical College and Institute of Dentistry

**Keywords:** cerebrovascular accident, stroke, knowledge, undergraduate, assessment, thrombolysis, pakistan, interns, survey, hemiplegia

## Abstract

Introduction:

Stroke is the leading cause of neurological disability in the world. In Pakistan, house officers (HOs) are usually the first contact for a stroke patient in the emergency department. Sometimes they need to make quick decisions regarding diagnosis and management without specialist supervision. Thousands of current final year MBBS (Bachelor of Medicine, Bachelor of Surgery) students will be performing the duties of HOs soon. This study documents the knowledge and confidence levels of final year students and HOs in Pakistan regarding basic facts related to initial diagnosis and management of stroke.

Materials and Methods:

A questionnaire was developed using two standard textbooks of medicine and current stroke guidelines of the American Heart Association. The pre-tested self-administered questionnaire was distributed among 800 final year MBBS students and HOs in 14 medical colleges and hospitals in four different cities. The response rate was 88.5%. Data analysis was done using SPSS V.21. The CMH Lahore Medical College Ethics Review Committee approved this project.

Results:

Respondents included medical students (n=496) and HOs (N= 212); most were female (n = 452, 63.9%). Of these, 31.4% had managed or assisted in the management of a patient with a stroke and had a higher confidence level in its diagnosis (p< 0.001) and management (p <0.001). Having a family member with stroke was associated with higher confidence in the diagnosis of stroke (p < 0.05) but not with confidence in its management (p = 0.41). Most correctly defined stroke (60.6%), identified the CT scan as the initial diagnostic modality (88.1%), knew the dosage of aspirin (64.9%), knew the time limit for thrombolysis (67.4%), and were familiar with the risk of deep vein thrombosis in immobilized stroke patients (85.4%). Less than half (44.5%) chose tissue plasminogen activator (t-PA) as the preferred initial intervention for acute ischemic stroke.

Conclusion:

This multicenter survey shows that the knowledge and confidence of medical students and HOs in Pakistan regarding initial diagnosis and management of stroke are inadequate in most domains. There is a need to improve the medical training for stroke in emergency departments for optimal outcomes. Public education campaigns about stroke should be conducted to increase the general awareness of the population about the prevention, signs, symptoms, and emergency steps to be taken when encountering a case of stroke.

## Introduction

Stroke is a cerebral deficit due to vascular blockage (ischemic stroke) or leakage (hemorrhagic stroke) lasting more than 24 hours [1]. It is the second leading global cause of mortality, causing an estimated 6.7 million deaths worldwide in 2012 [2]. Most deaths from stroke are in the low and middle-income countries. Considering its huge burden on health, it follows that the level of knowledge about stroke and its initial diagnosis and management, both in the general population and physicians, should proportionately match its prevalence.

Stroke is an emergency condition, and presentation of a case in the emergency department does not wait for regular physician practice hours. In the current health care system of Pakistan, the first physicians to attend these patients are usually the HOs, who are interns having graduated from medical college in the past year. They are sometimes on call for 24-32 hours at a time. Currently, there are 96 accredited medical schools in Pakistan with thousands of medical students ready to begin their careers as HOs in less than a year [3]. These students will be attending many stroke cases in emergency departments all around Pakistan.

Early diagnosis and management are important in treating a stroke case. The effectiveness of thrombolytic therapy in ischemic stroke is time-dependent (most effective within 4.5 hours since onset) [4]. Therefore, it is essential that HOs be trained and be confident in their knowledge about the immediate diagnosis and management of stroke so that the initial "golden hour" is not wasted.

The aim of this study was to document the knowledge and confidence levels of final year medical students and HOs in Pakistan regarding basic facts about the initial diagnosis and management of stroke.

## Materials and methods

Study approval was obtained from the Combined Military Hospital (CMH) Lahore Medical College Ethics Review Committee. A cross-sectional survey design was used. The data collection form had three parts. The first part was the informed consent form, which described the details of the research project, its possible impact, and assurance of anonymity of the data. The second part had demographic details, including the institutional affiliation, age, gender, and work experience of the HOs. In addition, it had questions related to the level of confidence in the diagnosis and management of a case with stroke and whether the respondent had a family member with stroke. The third part consisted of fifteen questions addressing diagnosis, investigations, management, pharmacotherapy, and complications of a stroke.

The questions were prepared in the single best choice multiple choice question (MCQ) format. The main stem was followed by four possible choices and respondents had to choose the most appropriate answer. Neurology chapters of two standard textbooks of medicine widely used by the undergraduate medical students in Pakistan were consulted for the construction of these questions. These included Davidson's Principles and Practice of Medicine, 22nd Edition, and Kumar and Clark's Clinical Medicine, 8th Edition [5-6]. In addition, the American Heart Association/American Stroke Association Guidelines for the Early Management of Patients with Acute Ischemic Stroke publication was also consulted [4]. Two professors of neurology working in two different medical schools independently reviewed the MCQs as subject experts for content validity. The questionnaire was constructed as a test based on the best choice question format with only one correct answer and three wrong answers, rather than a Likert scale type questionnaire. Therefore, an exploratory factor analysis was not performed.

The questionnaire was pilot tested on 15 HOs of CMH Lahore. From this pilot, the survey time was estimated to be about 10 minutes. Questions were well understood and only minor changes in clarity and language were needed for the final survey. The final questionnaire was administered to 850 final year medical students and HOs in 13 different colleges and medical institutes in Lahore, Rawalpindi, Islamabad, and Quetta. The lead author, co-authors, or a representative who was briefed in advance collected the data. The response rate was 88.9 %. Forty-eight forms were discarded (incomplete or multiple entries) and the data from 708 forms was entered. 

Data were analyzed in SPSS V 21 (IBM, Chicago, Illinois). Frequencies and percentages were calculated for categorical variables, including demographics of respondents and responses to questions related to stroke. Mean (minimum-maximum) scores were calculated for answers on the questionnaire assessing knowledge related to stroke. Correct answers were coded as 1 and incorrect answers as 0.

Knowledge item analysis was conducted to determine the difficulty level of the stroke knowledge questionnaire. It was determined by following parameters: a) Item difficulty, b) discrimination coefficients, c) distracter analysis, and d) readability analysis as assessed by the Flesch reading ease rating and the Flesch-Kincaid grade.

Pearson Chi-Square was used to analyze associations between having a family member with a stroke and participants’ confidence in the diagnosis and management of a stroke. Similar associations were analyzed for having managed a case of stroke and its association with participants’ confidence in the diagnosis and management of a stroke. Statistical differences on mean scores obtained on the knowledge of stroke questionnaire were tested between the final year medical students and HOs as well as past experiences with management of a stroke and having a family member with a stroke. These associations were tested by running the t-test for independent samples.

The item difficulty index (P) was calculated by the formula P = R/T, where R is the number of correct responses and T is the total number of responses. Increasing scores on the item difficulty index correspond with the low difficulty level of the item. For the stroke questionnaire employed in this study, items with four alternative items should have an optimum difficulty level of 0.625. For the present study, values of (P) between 0.20 and 0.90 are considered acceptable; however, items scoring < .20 can be added as easy “warm-up” questions and those scoring > .90 might be necessary to distinguish between average and top performers.

The point-biserial correlation was used to analyze the strength of association between each item and overall scores on the stroke knowledge questionnaire. The point-biserial correlation coefficient ranges from −1.00 to 1.00. Greater values correspond with a higher discriminating power of the item. A coefficient value of 0.20 or higher was considered acceptable in this study [7]. The percentage of participants selecting particular distracters (incorrect choices) was analyzed for each item in detail. Finally, suggestions were provided to improve the questionnaire, keeping the difficulty levels and discrimination coefficients in context.

## Results

There were 708 valid forms from 496 (70.1%) final year medical students and 212 (29.9%) HOs. There were 256 (36.1%) males and 452 (63.9%) females. Approximately a quarter of the students and HOs had a family member with stroke (144, 20.3%), and 222 (31.4%) students and HOs had managed or assisted in the management of stroke cases.

Having a family member with a stroke was associated with a significantly higher confidence in the diagnosis of stroke (Chi-square 7.83, p = 0.02) but not with confidence in its management (Chi-square= 1.7, p = 0.41). Students or HOs who had assisted in the management of stroke cases had a higher confidence level in its diagnosis (Chi-square= 69.25, p < 0.001) and management (Chi-square= 71.88, p < 0.001). These results are displayed in Table 1. 

**Table 1:** Correlates of Confidence in the Diagnosis and Management of Stroke *Missing values = 43-76 participants did not respond to these questions.

Variables		Confidence in Diagnosis of Stroke			Confidence in Management of Stroke		
		Very confident	Somewhat confident	Not confident	Very confident	Somewhat confident	Not confident
Family member with stroke	Yes	33 (23.4%)	94 (66.7%)	14 (9.9%)	22 (15.6%)	59 (41.8%)	60 (42.6%)
	No	119 (23.3%)	291 (56.9%)	101 (19.8%)	56 (11.4%)	215 (43.8%)	220 (44.8%)
Assisted in management of a case of stroke	Yes	82 (37.4%)	131 (59.8%)	6 (2.7%)	39 (18.1%)	132 (61.4%)	44 (20.5%)
	No	72 (16.1%)	264 (59.2%)	110 (24.7%)	41 (9.5%)	150 (34.9%)	239 (55.6%)

Of the participants, 60.6% knew the definition of stroke, 88.1% knew the best initial test for it, but only 25.9% knew the most accurate stroke investigations. The best initial therapy for ischemic stroke was known to just 44.5% and only 58.1% knew the first step in the management of hemorrhagic stroke. However 75.7% knew that oral aspirin should be administered within 24-48 hours of ischemic stroke. A mere 31.5% were aware of when to prescribe statins in a patient with ischemic stroke. A larger percentage knew that thrombolysis in ischemic stroke is most effective within four hours (67.4%) and that, in a patient presenting with ischemic stroke already taking aspirin, clopidogrel should be added to the prescription (60.6%). Most of the respondents knew that lowering and maintaining the blood pressure lowers the risk of stroke recurrence (70.3%), that hemorrhagic stroke is an absolute contraindication to thrombolytic therapy (70.6%), and that deep venous thrombosis (DVT) has to be monitored in an immobilized patient (85.4%). The detailed results are shown in Table 2.

**Table 2:** Item-wise Response Distribution on Stroke Knowledge Test *Correct answer † 2-12 participants did not respond to these questions.

	A	B	C	D
	n (%)	n (%)	n (%)	n (%)
A stroke is a focal deficit of brain function, most commonly hemiplegia, with or without signs of focal higher cerebral dysfunction, lasting:	149 (21.3%)	96 (13.7%)	425* (60.6%)	31 (4.4%)
The first investigation of choice in a patient suspected of having acute stroke is?	622* (88.1%)	63 (8.9%)	14 (2.0%)	7 (1.0%)
The initial diagnostic test of choice in a case of stroke helps in:	608* (86.2%)	15 (2.1%)	39 (5.5%)	43 (6.1%)
The most accurate diagnostic test for detecting ischemic stroke?	88 (12.6%)	181* (25.9%)	315 (45.0%)	116 (16.6%)
The best initial therapy for a non-hemorrhagic stroke patient presenting within 4.5 hrs from the start of symptoms is:	247 (35.0%)	112 (15.9%)	32 (4.5%)	314* (44.5%)
The first step of management in a patient of hemorrhagic stroke should include which of the following?	409* (58.1%)	33 (4.7%)	125 (17.8%)	137 (19.5%)
Which of the following oral medicines should be administered within 24-48 hours of the onset of stroke?	125 (17.8%)	23 (3.3%)	532* (75.7%)	23 (3.3%)
What is the dose of aspirin to be given in ischemic stroke?	95 (13.5%)	98 (13.9%)	457* (64.9%)	54 (7.7%)
Statins should be prescribed to a patient with a stroke if the serum total cholesterol is:	85 (12.2%)	219* (31.5%)	181 (26.0%)	211 (30.3%)
Thrombolysis in an ischemic stroke is the most effective when administered:	471* (67.4%)	60 (8.6%)	149 (21.3%)	19 (2.7%)
A 40-year-old patient presenting with an acute stroke is taking aspirin. What medicine should be added to his/her prescription?	185 (26.4%)	33 (4.7%)	58 (8.3%)	425* (60.6%)
The risk of stroke recurrence can be significantly reduced by:	17 (2.4%)	136 (19.4%)	55 (7.8%)	493* (70.3%)
Which of the following is an absolute contraindication to thrombolytic therapy?	496* (70.6%)	116 (16.5%)	28 (4.0%)	63 (9.0%)
Development of what serious condition would you be concerned about the most in patients immobilized due to acute stroke?	36 (5.1%)	49 (7.0%)	17 (2.4%)	599* (85.4%)
The most important contraindication to anticoagulation is:	53 (7.6%)	496* (71.1%)	60 (8.6%)	89 (12.8%)

Mean score ± standard deviation (min-max) obtained on the stroke scale was 9.56 ± 2.7 (1-15). According to the t-test for independent samples, HOs scored higher on the stroke questionnaire as compared to final year medical students (t = 9.63, p < .001). Those participants who had previously managed a case of stroke scored higher on the stroke questionnaire as compared to their counterparts (t = 3.43, p < 0.005). However, having a family member with stroke did not yield any significant association with the mean scores on the stroke questionnaire (p = .968).

The Flesch reading ease rating for the knowledge test was 54.6 and the Flesch-Kincaid grade was 7.9, indicating that the items should be understandable to students of 13-15 years of age [8].

Compared to medical students, a higher percentage of HOs answered correctly on most of the items. There was no significant difference in the proportion of HOs and medical students answering correctly on Items 7, 8, 9, and 10. The percentage of missing responses ranged from 0.3 % - 1.4% which was not significantly problematic. Detailed results have been given in Table 3.

**Table 3:** Item-wise Response Distribution on Stroke Knowledge Test Based on Participants' Career Status

Questions		Participant Status				P-value	Missing Responses
		Final-year Students		House Officers			
		Frequency (n)	Percentage (%)	Frequency (n)	Percentage (%)		
Question 1	Incorrect	211	42.5%	72	34.0%	.033	7 (1%)
	Correct	285	57.5%	140	66.0%		
Question 2	Incorrect	80	16.1%	6	2.8%	< .001	2 (.3%)
	Correct	416	83.9%	206	97.2%		
Question 3	Incorrect	91	18.3%	9	4.2%	< .001	3 (.4%)
	Correct	405	81.7%	203	95.8%		
Question 4	Incorrect	380	76.6%	147	69.3%	.042	8 (1.1%)
	Correct	116	23.4%	65	30.7%		
Question 5	Incorrect	292	58.9%	102	48.1%	.008	3 (.4%)
	Correct	204	41.1%	110	51.9%		
Question 6	Incorrect	235	47.4%	64	30.2%	< .001	4 (.6%)
	Correct	261	52.6%	148	69.8%		
Question 7	Incorrect	130	26.2%	46	21.7%	.203	5 (.7%)
	Correct	366	73.8%	166	78.3%		
Question 8	Incorrect	185	37.3%	66	31.1%	.116	4 (.6%)
	Correct	311	62.7%	146	68.9%		
Question 9	Incorrect	343	69.2%	146	68.9%	.940	12 (1.7%)
	Correct	153	30.8%	66	31.1%		
Question 10	Incorrect	164	33.1%	73	34.4%	.724	9 (1.3%)
	Correct	332	66.9%	139	65.6%		
Question 11	Incorrect	251	50.6%	32	15.1%	< .001	7 (1%)
	Correct	245	49.4%	180	84.9%		
Question 12	Incorrect	162	32.7%	53	25.0%	.042	7 (1%)
	Correct	334	67.3%	159	75.0%		
Question 13	Incorrect	181	36.5%	31	14.6%	< .001	5 (.7%)
	Correct	315	63.5%	181	85.4%		
Question 14	Incorrect	97	19.6%	12	5.7%	< .001	7 (1%)
	Correct	399	80.4%	200	94.3%		
Question 15	Incorrect	182	36.7%	30	14.2%	< .001	10 (1.4%)
	Correct	314	63.3%	182	85.8%		

As detailed in Table 4, all of the items had difficulty levels (P) and discrimination coefficient values in acceptable ranges, except item 9, which had an optimum difficulty index but a non-significant discriminatory coefficient. It yielded only 219 (30.9%) correct answers and the distracter analysis revealed that stem D with 30.3% responses followed with stem C having 26% responses. 

**Table 4:** Item-wise Difficulty Level of Stroke Knowledge Test *p = > 0.05 ‡p < 0.001

Question	Item Difficulty	Point Biserial Correlation Coefficient	Distracter Analysis
1: A stroke is a focal deficit of brain function, most commonly hemiplegia, with or without signs of focal higher cerebral dysfunction, lasting:	.60	.50‡	Distracter A “More than 12 hours” was selected by 21.3%.
2: The first Investigation of choice in a patient suspected of having an acute stroke is?	.88	.33‡	
3: The initial diagnostic test of choice in a case of stroke helps in:	.86	.44‡	
4: The most accurate diagnostic test for detecting ischemic stroke?	.26	.24‡	Distracter C “Contrast Computed Tomography (CT) scan of head” was selected by 44.5%
5: The best initial therapy for a non-hemorrhagic stroke patient presenting within 4.5 hrs from start of symptoms is:	.44	.45‡	Distractor A “Aspirin” selected by 34.9%
6: The first step of management in a patient with hemorrhagic stroke should include which of the following?	.58	.50‡	
7: Which of the following oral medicines should be administered within 24-48 hours of onset of stroke?	.75	.29‡	
8: What is the dose of aspirin to be given in ischemic stroke?	.65	.40‡	
9: Statins should be prescribed to a patient with stroke if the serum total cholesterol is:	.31	.02*	This question does not correlate well with the overall score, but it has an optimum difficulty level. A total of 219 (30.9%) gave correct answers and distracter analysis revealed that distracter D had 30.3% responses, followed with distracter C having 26% responses.
10: Thrombolysis in Ischemic stroke is most effective when administered:	.67	.48‡	Distracter C “Within 12 hrs of onset of symptoms” by 21%
11: A 40-year-old patient presenting with acute stroke is taking aspirin. What medicine should be added to his prescription?	.60	.54‡	Distracter A “Streptokinase” by 26%
12: The risk of stroke recurrence can be significantly reduced by:	.70	.38‡	Distracter B "Regular Exercise" by 19.2%. Are students confusing it as an intervention to decrease BP? Reword to “The risk of stroke recurrence can be *BEST* reduced by”
13: Which of the following is an absolute contraindication to thrombolytic therapy?	.70	.55‡	
14: Development of what serious condition would you be concerned about the most in patients immobilized due to acute stroke?	.85	.48‡	
15: The most important contraindication to anticoagulation is:	.70	.58‡	

The complete questionnaire with the answer key is displayed in Table 5

**Table 5:** Complete Questionnaire for HOs

Please select and circle only one answer for each question.					
Question	Stem A	Stem B	Stem C	Stem D	Correct Answer
1) A stroke is a focal deficit of brain function, most commonly hemiplegia, with or without signs of focal higher cerebral dysfunction, lasting:	More than 12 hrs	Less than 12 hrs	More than 24 hrs	Less than 24 hrs	C
2) The first Investigation of choice in a patient suspected of having an acute stroke is?	Computed tomography (CT) scan	Magnetic resonance imaging (MRI)	X-ray head	Lumbar puncture (LP)	A
3) The initial diagnostic test of choice in a case of stroke helps in:	Distinguishing between haemorrhagic and ischaemic stroke	Identification of post-stroke depression	Localizing the aneurysm	Preventing further haemorrhage.	A
4) The most accurate diagnostic test for detecting ischemic stroke?	Non-diffusion weighted magnetic resonance imaging (MRI)	Diffusion-weighted magnetic resonance imaging (MRI)	Contrast computed tomography (CT) scan of head	Non-contrast computed tomography (CT) scan of head	B
5) The best initial therapy for a non-hemorrhagic stroke patient presenting within 4.5hrs from start of symptoms is:	Aspirin	Heparin	Warfarin	Tissue plasminogen activator (tPA)	D
6) The first step of management in a patient with haemorrhagic stroke should include which of the following?	Lower blood pressure (BP)	Give statins	Give intravenous fluids	Give intravenous 5000 Units of heparin	A
7) Which of the following oral medicines should be administered within 24-48 hours of the onset of stroke?	Prednisone	Gabapentin	Aspirin	Fluoxetine	C
8) What is the dose of aspirin to be given in ischaemic stroke?	75 mg daily	300 mg stat only	300 mg stat & 75 mg daily	300 mg stat & 300 mg daily.	C
9) Statins should be prescribed to a patient with stroke if the serum total cholesterol is	More than 2.5 mmol/L (100 mg/dL)	More than 3.5 mmol/L (135 mg/dL)	More than 4 mmol/L (155 mg/dL)	More than 5 mmol/L. (195 mg/dL)	B
10) Thrombolysis in ischaemic stroke is most effective when administered	Within 4.5 hrs after onset of symptoms	After 4.5 hrs of onset of symptoms	Within 12 hrs of onset of symptoms	After 12 hrs of onset of symptoms.	A
11) A 40-year-old patient presenting with acute stroke is taking aspirin. What medicine should be added to his/her prescription?	Streptokinase	Vit B-12	Propranolol	Clopidogrel	D
12) The risk of stroke recurrence can be significantly reduced by:	Good sleep	Regular exercise	Subcutaneous heparin	Reduction and maintenance of blood pressure	D
13) Which of the following is an absolute contraindication to thrombolytic therapy?	Intracerebral haemorrhage (ICH)	Pregnancy	Age > 50 years	Major surgery within the last 1 year.	A
14) The development of what serious condition would you be concerned about the most in patients immobilized due to acute stroke?	Depression	Dementia	Urinary tract infection	Deep vein thrombosis (DVT)	D
15) The most important contraindication to anticoagulation is:	Epilepsy	Haemorrhagic stroke	Deep vein thrombosis (DVT)	Pregnancy	B

## Discussion

In our sample of 708 respondents from 13 different medical colleges in Pakistan, only 31.4% of the participants had ever managed or assisted in the management of a stroke patient. However, those who had been involved in stroke management were more confident in both diagnosis and management. More than half (55.5%) were not aware of the recommended initial therapy for ischemic stroke and 41.9% were unaware of the initial steps involved in the management of hemorrhagic stroke. Most participants (68.5%) were also unaware about when to start a patient of ischemic stroke on statins. However, the majority had knowledge about the use of aspirin (75.7%), timely thrombolysis (67.4%), maintaining blood pressure for preventing recurrences (70.3%), and the risk of DVT in immobilized patients (85.4%). HOs reported better knowledge related to stroke than final year students. Having managed a case of stroke was associated with better knowledge of stroke. However, having a family member with stroke did not yield any significant difference.

A previous survey performed on family physicians in Pakistan to assess knowledge about stroke care yielded deficiencies similar to our study. Noticeably, less than half correctly identified all five symptoms of stroke (46%) and prescribed statins for secondary stroke prevention (44%), and only two-thirds adequately treated hypertension [9]. It is not surprising, therefore, that more than 35% of strokes and more than 50% of transient ischemic attacks (TIAs) in Pakistan are misdiagnosed [10]. This might lead to inadequate or even harmful treatment, poor patient outcomes, and decreased patient satisfaction.

Surveys from all around the globe have revealed that the generic knowledge about stroke in the general population is generally inadequate in both the developed [11-13] and developing countries [14-16]. In a survey carried out in six Gulf countries, the majority of patients were not even aware of the term "stroke" [14]. A survey carried out in Pakistani high schools in a single district showed that most of the respondents were unaware of stroke risk factors, symptoms, and did not know what to do in case of an acute stroke [17]. There is a need for a regular and massive public education campaign to improve awareness and, therefore, early intervention in stroke.

Current data about the countrywide prevalence of stroke in Pakistan is not available, but according to an estimate of a subpopulation, 4.8% of its population might have a stroke annually [18]. This is equivalent to approximately 8.7 million stroke patients in Pakistan. Of those affected by a stroke, 63% develop complications, 89% become dependent, and in Pakistan, unfortunately, 7-20% die [10]. It also adds a huge burden on the healthcare system, families, and the economy. Given the fact that early diagnosis and timely intervention cannot only prevent death, but also avoid debilitation and complications, adequate treatment of stroke becomes very significant in a society where whole families often rely on a single person for their sustenance.

Medical education in Pakistan presents a wide spectrum of undergraduate and postgraduate training and is not standardized across the country. At one end are the public medical colleges, which provide a variety and a huge number of cases in all disciplines of medicine and surgery for training and exposure, but might lack adequate facilities, resources, or the workforce to deal with them and utilize their educative potential properly. On the other end are private medical institutes, which might have state-of-the-art facilities and dedicated faculty willing to teach students, but usually lack the variety of clinical cases due to high treatment costs and the prevalence of poverty in the general population.

Therefore, it is necessary that medical personnel in general and HOs, in particular, be trained in a standardized manner, through focused lectures, bedside teaching sessions, and interactive workshops, in the management of prevalent and potentially disabling neurological emergencies, such as stroke, meningitis, acute paraplegia or hemiplegia, etc. It is also important to target neurological topics to better prepare and help the medical workforce in assisting their future and current patients, respectively. Public education campaigns to raise awareness about prevention, initial symptoms, and emergency measures in the case of a stroke should be held. The Pakistan Medical and Dental Council (PMDC), the College of Physicians and Surgeons of Pakistan (CPSP), and the Higher Education Commission (HEC), in collaboration with national societies (Pakistan Society of Neurology, Pakistan Society of Physical Medicine and Rehabilitation, and Pakistan Stroke Society), should devise a national policy on stroke, targeting the physicians and the public. It should focus on teaching the undergraduate medical students the basic facts in recognizing a stroke and training the HOs, postgraduate residents, and general physicians in the correct diagnosis and emergency management of acute stroke. In addition, there is a need to conduct a countrywide epidemiological survey on stroke. There is also an urgent need to establish departments of neurology and rehabilitation medicine at all major public hospitals and medical schools. While the neurologist ensures timely diagnosis and management of acute stroke, the rehabilitation physician coordinates the post-stroke rehabilitation and smooth transition back to everyday life.

There are certain limitations of this study worth mentioning. Although this was a large sample size from 13 different medical colleges and hospitals, it still represents a fraction of the estimated 25,000 medical students in 97 medical colleges across the country. In addition, we did not correlate the confidence levels and knowledge about the basic facts of stroke with the work experience of HOs. Moreover, departments of neurology are not established in each medical college and might be a contributory factor towards inadequate knowledge in certain domains of stroke diagnosis and management.

Although this study was conducted in Pakistan, it is likely that similar problems and gaps in stroke care exist in most of the countries in the developing world. We propose similar or more extensive surveys targeting medical students, internees, residents, and physicians in other developing countries. This would help to highlight the deficits and recommend solutions to improve the quality of stroke care and reduce the global burden of this preventable disease.

## Conclusions

Considering the worldwide prevalence, morbidity, and mortality of stroke and the fact that the current final year medical students and HOs will ultimately face the challenge of managing stroke cases eventually, it is evident from this study that both of these groups are not equipped with some essential, life-saving information. The results also suggest that some areas, such as initial therapeutic measures, require more attention than the others in order to save lives and prevent complications. It is important that all aspects of stroke management in general and some, in particular, be targeted to make sure that every physician is well-versed with at least the basic steps to take when facing a case of stroke. On a wider scale, public education campaigns about stroke should be conducted to increase the general awareness of the population about the prevention, signs, symptoms, and emergency steps to be taken when encountering a case of stroke.
